# COVID-19 is moving to high-density, poor residential areas in Metropolitan Manila, Philippines

**DOI:** 10.5365/wpsar.2020.11.2.003

**Published:** 2021-01-08

**Authors:** Eumelia P. Salva Villarama, Edmundo B. Lopez, Ana Ria Sayo, Xerxes Seposo, Koya Ariyoshi, Chris Smith

**Affiliations:** a San Lazaro Hospital, Manila, Philippines.; b School of Tropical Medicine and Global Health, Nagasaki University, Nagasaki, Japan.; c Institute of Tropical Medicine, Nagasaki University, Nagasaki, Japan.; d Faculty of Infectious and Tropical Diseases, London School of Hygiene and Tropical Medicine.

**Figure 1:**
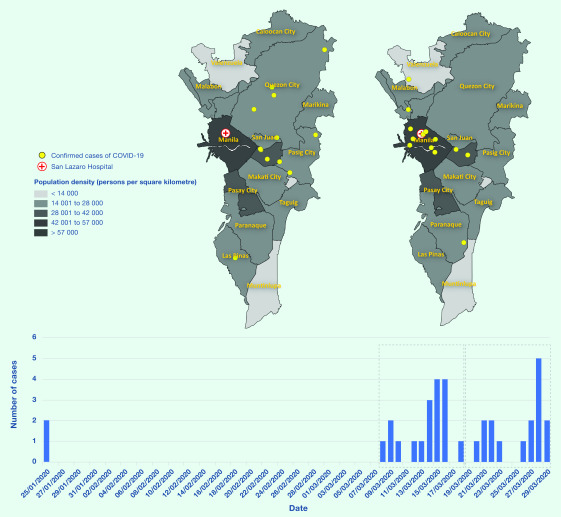
Timeline of cases with confirmed COVID-19 admitted to San Lazaro Hospital, from 25 January to 29 March 2020. Maps show the residence of patients in the National Capital Region (Metropolitan Manila) admitted during 8–18 March (left) and 19–29 March (right)

We note three successive waves of coronavirus disease 2019 (COVID-19) cases in the National Capital Region (Metropolitan Manila), Philippines: the first was from imported cases among Chinese nationals; the second was from infections among Filipinos residing in less densely populated areas; and the third was from infections among Filipinos residing in high-density, poor areas. We support these observations with data from San Lazaro Hospital, the national infectious diseases hospital, which serves a low-income population in Manila City, the most densely populated city within Metropolitan Manila (**Fig. 1**).

[**insert** Figure 1]

The first two confirmed cases of COVID-19 in the Philippines were among Chinese nationals on vacation, both of whom were admitted to San Lazaro Hospital on 25 January 2020, with confirmation on 31 January and 1 February. (*1*) A third imported case from China was confirmed on 3 February 2020. (*2*) Despite concerns that all three individuals had travelled widely within the Philippines, no secondary infections arising from these cases were confirmed.

The next person with confirmed COVID-19 was admitted to San Lazaro Hospital more than one month later, on 8 March. During the following 10 days, a further 17 confirmed cases were reported at the hospital. In contrast to the first individuals with confirmed COVID-19, these individuals were all Filipinos, with seven reporting recent travel to areas affected by COVID-19. None of these patients resided in the densely populated catchment area of the hospital. From 19 to 29 March, a further 16 cases were confirmed at the hospital. In contrast to the previous wave, all patients except for one resided in Manila City, with only one reporting a significant history of international travel.

The occurrence of confirmed COVID-19 in  Manila City is concerning given that it has an estimated population density of 71 263 persons per square kilometre. In the Philippines overall, there were 9223 confirmed cases as of 3 May. (*2*) The true number of cases is likely to be much higher given that until late March testing was conducted by only one laboratory in the country. Significant community transmission cannot be excluded due to the lack of laboratory surveillance data. The establishment of subnational laboratories across the Philippines, including at San Lazaro Hospital, is timely and welcome. In Manila City, increased community testing and monitoring of individuals presenting to hospitals with respiratory symptoms could detect increased COVID-19 transmission.

At-home isolation for 14 days is now recommended for people with mild COVID-19; (*3*) however, for people living in high-density or slum areas, it will be challenging to ensure that they are able to adequately isolate to avoid further transmission. The planned establishment of designated isolation facilities and expansion of testing should help reduce community transmission. (*4*)

A surge of severe or high-risk cases in Manila City is likely to put enormous pressure on health-care facilities, which are already experiencing significant infections among health-care workers and shortages of personal protective equipment. Bed shortages may become more acute if other infectious disease outbreaks occur, such as measles, dengue or leptospirosis.

Luzon island has been under community quarantine since 15 March 2020. (*5*) People living in high-density areas, such as Manila City, are likely to be more vulnerable to the negative consequences of community quarantine, such as economic difficulties, food insecurity and domestic violence. It is hoped that the quarantine measures will flatten the epidemic curve and result in fewer overall infections, but they may be difficult to sustain for a long period.
